# Impact of relative cisplatin dose to skeletal muscle mass on adverse events in patients with head and neck cancer undergoing chemoradiotherapy

**DOI:** 10.1093/oncolo/oyae167

**Published:** 2024-07-09

**Authors:** Satoshi Suzuki, Tomoya Yokota, Akifumi Notsu, Satoshi Hamauchi, Yusuke Onozawa, Kunihiro Fushiki, Kotoe Oshima, Takeshi Kawakami, Takahiro Tsushima, Hirofumi Yasui, Hirofumi Ogawa, Tsuyoshi Onoe, Keisuke Kawatani, Kentaro Yamazaki

**Affiliations:** Division of Gastrointestinal Oncology, Shizuoka Cancer Center, Shizuoka, Japan; Division of Gastrointestinal Oncology, Shizuoka Cancer Center, Shizuoka, Japan; Department of Clinical Research Center, Shizuoka Cancer Center, Shizuoka, Japan; Division of Gastrointestinal Oncology, Shizuoka Cancer Center, Shizuoka, Japan; Division of Medical Oncology, Shizuoka Cancer Center, Shizuoka, Japan; Division of Gastrointestinal Oncology, Shizuoka Cancer Center, Shizuoka, Japan; Division of Gastrointestinal Oncology, Shizuoka Cancer Center, Shizuoka, Japan; Division of Gastrointestinal Oncology, Shizuoka Cancer Center, Shizuoka, Japan; Division of Gastrointestinal Oncology, Shizuoka Cancer Center, Shizuoka, Japan; Division of Gastrointestinal Oncology, Shizuoka Cancer Center, Shizuoka, Japan; Division of Radiation and Proton Therapy Center, Shizuoka Cancer Center, Shizuoka, Japan; Division of Radiation and Proton Therapy Center, Shizuoka Cancer Center, Shizuoka, Japan; Department of Diagnostic imaging, Shizuoka Cancer Center, Shizuoka, Japan; Division of Gastrointestinal Oncology, Shizuoka Cancer Center, Shizuoka, Japan

**Keywords:** head and neck squamous cell carcinoma, cisplatin, skeletal muscle mass, adverse events, chemoradiotherapy

## Abstract

**Background:**

Chemoradiotherapy (CRT) with high-dose cisplatin (CDDP) is the standard treatment for locally advanced head and neck squamous cell carcinoma (HNSCC). Although dosing is based on body surface area (BSA), some patients experience CDDP-related adverse events (AEs). We aimed to evaluate the impact of relative CDDP dose to skeletal muscle mass (SMM) on chemotherapy-associated AEs in patients with HNSCC undergoing CRT with high-dose CDDP.

**Materials and Methods:**

We retrospectively analyzed data of patients who underwent CRT with high-dose CDDP (80-100 mg/m^2^, triweekly) between 2010 and 2023. SMM was measured as the cross-sectional muscle area at the third cervical vertebra (C3-SMM) using computed tomography; the skeletal muscle index (SMI) was defined as SMM normalized by squared height. The CDDP index was defined as the prescribed CDDP dose divided by SMI. CDDP-related AEs were assessed during the first cycle using Common Terminology Criteria for Adverse Events v5.0.

**Results:**

Overall, 306 patients were identified. The CDDP index cutoff value best associated with grade ≥ 3 AEs was 10.312. Grade ≥ 3 hematological toxicities exhibited stronger association with high CDDP index value than with low CDDP index value (26.9% vs 16.3%, *P* = .033). Multivariate analysis revealed that high CDDP index value and creatinine clearance < 70 mL/minute were predictive factors for grade ≥ 3 AEs (odds ratio [OR] 2.55, *P* = .008; OR 3.68, *P* = .002, respectively).

**Conclusion:**

The CDDP index based on C3-SMM was an independent predictive factor for grade ≥ 3 CDDP-related AEs. C3-SMM is potentially more useful than BSA for determining the optimal CDDP dose in patients with HNSCC.

## Teaser

Chemoradiotherapy with high-dose cisplatin is the standard treatment for locally advanced head and neck squamous cell carcinoma (HNSCC). This study evaluated the effect of relative cisplatin dose to skeletal muscle mass on chemotherapy-associated adverse events in patients with HNSCC.

**Implications for practice**

Body composition has emerged as an important predictive factor for chemotherapy-related toxicity in various solid tumors. We defined the relative cisplatin (CDDP) dose to skeletal muscle mass (SMM) at C3 level as CDDP index, and evaluated the impact of CDDP index on CDDP-related toxicities in patients with head and neck cancer who received chemoradiotherapy (CRT) with high-dose CDDP. High CDDP index was significantly associated with occurrence of grade ≥ 3 adverse events. Our result suggests that C3-SMM may be a useful biomarker to determine optimal CDDP dose in CRT for head and neck cancer.

## Introduction

Chemoradiotherapy (CRT) with concurrent high-dose cisplatin (CDDP) is the gold standard non-surgical treatment for patients with locally advanced head and neck squamous cell carcinoma (HNSCC).^1-3^ Although the prescribed CDDP dose is individually determined based on body surface area (BSA), its administration is commonly associated with adverse events (AEs), such as nausea, vomiting, renal insufficiency, ototoxicity, peripheral neuropathy, and cardiac overload due to large-volume infusion, leading to poor treatment compliance. BSA was used to estimate an appropriate starting dose for an anticancer drug, it seems that dose adjustment of CDDP based only on the BSA does not necessarily reduce toxicity. Therefore, there is an urgent need for a useful marker to determine the optimal CDDP dose to be administered.

Body composition has emerged as an important predictive factor for chemotherapy-related toxicity, as well as prognostic factor across various solid tumors, including breast, ovarian, prostate, colon, and esophageal cancers.^4-8^ Specifically, sarcopenia, a condition characterized by low skeletal muscle mass (SMM), is of interest.^7,9^ Several investigations have revealed that approximately half of patients with HNSCC have low SMM.^10-12^ Recent studies including patients with HNSCC have demonstrated associations between low SMM and an increased risk of chemotherapeutic toxicity.^10,13,14^ For instance, Wendrich et al. found that low SMM was an independent risk factor for dose-limiting chemotherapeutic toxicity in patients with locally advanced HNSCC underwent CRT.^10^ However, this report focused only on the correlation between low SMM and toxicity, but not taking the prescribed CDDP dose into consideration. Hence, the impact of the relative CDDP dose to SMM on AEs remains unclear.

In pharmacokinetics, approximately 10% of administered CDDP exists in a non-protein-bound form in the plasma, contributing to both antitumor activity and AEs.^15-17^ As CDDP is a hydrophilic drug, the non-protein-bound form of CDDP is distributed and metabolized within the lean body mass (LBM),^10,18-20^ with SMM being its largest component. Therefore, the volume of distribution for hydrophilic CDDP is highly dependent on the amount of SMM. However, CDDP clearance is expected to decrease with decreasing SMM, potentially leading to increased exposure and greater CDDP-related AEs. Therefore, SMM may be associated with the pharmacokinetics of CDDP in patients undergoing CRT.

We supposed that taking SMM and prescribed CDDP dose into consideration was useful to predict the occurrence of CDDP-related AEs. This study aimed to evaluate the impact of the relative CDDP dose to SMM on CDDP-related AEs in patients with HNSCC who underwent CRT with high-dose CDDP.

## Materials and methods

### Study design and patients

This study included consecutive 475 patients diagnosed with HNSCC who underwent concomitant CRT with high-dose CDDP (80-100 mg/m^2^) as definitive or postoperative treatment at Shizuoka Cancer Center (Shizuoka, Japan) between January 2010 and April 2023. Exclusion criteria for this study were prior treatment with platinum or radiotherapy for neck lesions, resection of the sternocleidomastoid muscle (SCM), clinical extra-nodal extension to the SCM, presence of double cancer, and existence of metal artifacts. This study was conducted in accordance with the ethical principles outline in the Declaration of Helsinki and was approved by the Institutional Review Board (IRB) of Shizuoka Cancer Center (IRB number: J2023-173-2023-1-3). Formal patient informed consent was waived due to the retrospective and anonymized fashion of this study.

### Measurement of SMM

**Figure 1.**
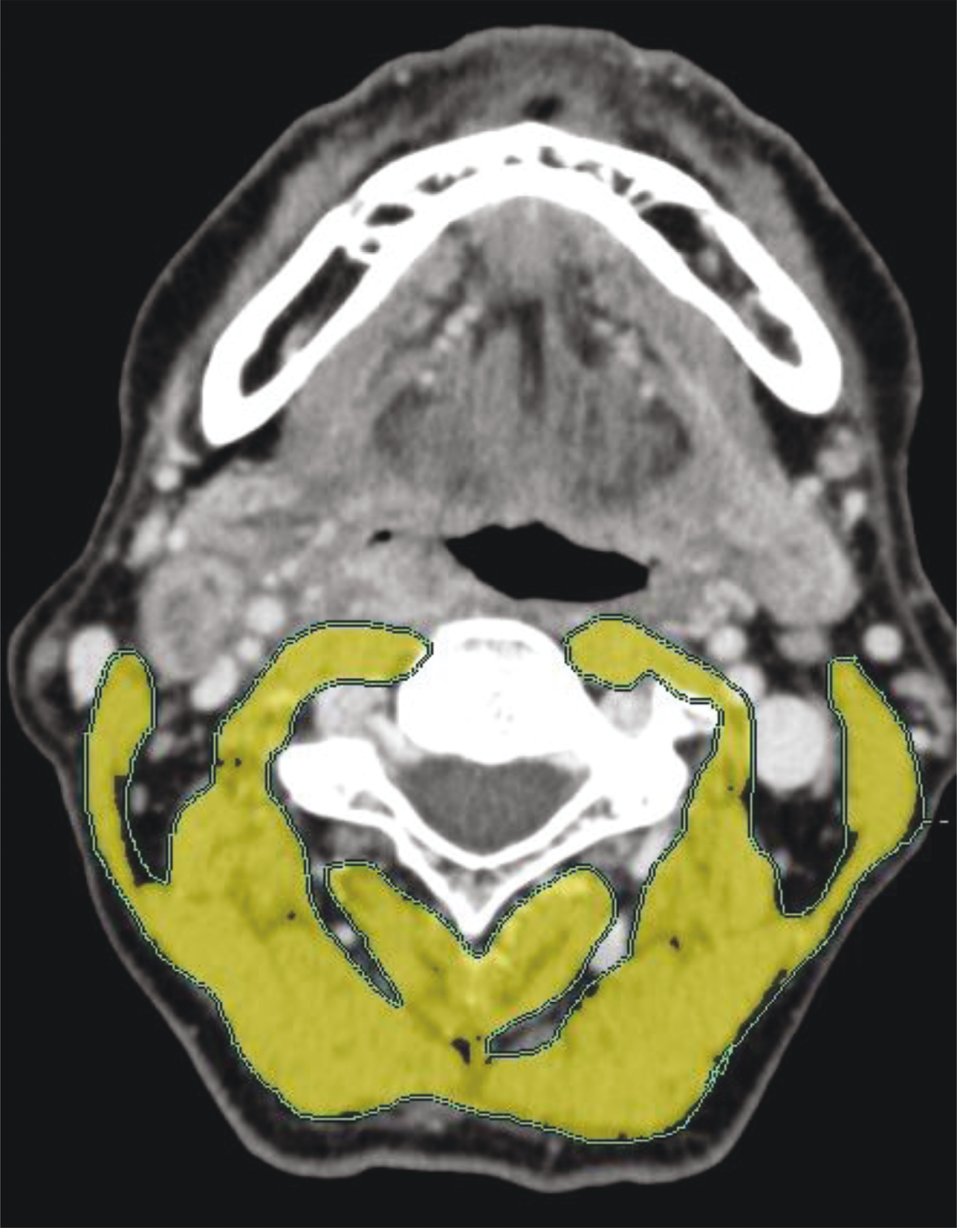
Skeletal muscle mass at the middle level of the third cervical vertebra (C3). The areas of the paravertebral muscle and both sternocleidomastoids at the C3 level were delineated and quantified using the SYNAPSE VINCENT software. Hounsfield Unit window from −29 to + 150 was used to accentuate paravertebral muscle and both sternocleidomastoids.


As part of radiotherapy planning, pretreatment imaging of head and neck lesions was performed using computed tomography (CT) in all patients. The total cross-sectional muscle area at the middle level of the third cervical vertebra (C3) was measured using the SYNAPSE VINCENT software (Fujifilm Co., Tokyo, Japan) within 2 months before treatment initiation (Figure 1). The Hounsfield Unit window from −29 to + 150 was used to quantify the paravertebral muscle and SCM areas. The skeletal muscle index (SMI) at the C3 level (C3-SMI) was defined as SMM normalized by squared height as follows^10^:

$$\begin{array}{l} C3-SMI\;(cm^{2}/m^{2})=C3-SMM\;(cm^{2})/height^{2}(m^{2}) \end{array}$$

Additionally, to account for the prescribed CDDP dose, we defined the CDDP index as the relative CDDP dose to C3-SMM as follows:


$$\begin{array}{l} CDDP\;index\;=\;prescribed\;CDDP\;dose\;(mg)/C3\text{-}SMI\;(cm^{2}/m^{2}) \end{array}$$

### Assessment of CDDP-related AEs

We assessed CDDP-related AEs during the first cycle of CDDP, including hematological toxicities (leukocytopenia, neutropenia, anemia, and thrombocytopenia), gastrointestinal toxicities (nausea, vomiting, and anorexia), nephrotoxicity, neuropathy (dysgeusia and ototoxicity), and infection. Since stomatitis and dermatitis might be considered as a radiation-induced toxicities, these were excluded from investigation. We checked blood test more than once a week. AEs were graded according to the Common Terminology Criteria for Adverse Events (CTCAE) version 5.0.

### Statistical analyses

Baseline variables considered as candidate predictive factors associated with grade ≥ 3 CDDP-related AEs were as follows: age (< 60 years vs 60 years ≤ < 70 years, vs ≥ 70 years), sex (male vs female), Eastern Cooperative Oncology Group (ECOG) performance status (PS) scale score (0 vs 1), treatment setting (postoperative vs definitive), Charlson risk index score (≥ 3 vs 2), serum albumin level, c-reactive protein (CRP) level, neutrophil-lymphocyte ratio (NLR), creatine clearance rate based on Cockcroft-Gault equation (CCR) (≥ 80 mL/minute vs 70 mL/minute ≤ < 80 mL/minute vs < 70 mL/minute), CDDP index value, and CDDP dose per BSA (80 mg/m^2^ vs 100 mg/m^2^). Serum albumin, CRP levels, NLR, and CCR were dichotomized with cutoff points set at 3.5 g/dL, 1 mg/dL, 3.7, and 80 mL/minute, respectively. The cutoff value for the CDDP index was determined based on receiver operating characteristic (ROC) curve, as described in the “Distribution of grade ≥3 CDDP-related AEs according to the CDDP index values” section. All statistical analyses were performed using EZR software (Saitama Medical Center, Jichi Medical University, Saitama, Japan). Continuous variables were compared between the groups using the Student’s *t* test, and categorical variables were compared between the groups using Fisher’s exact test. The predictive effect of variables on grade ≥ 3 CDDP-related AEs was evaluated using logistic regression analysis. An initial univariate Cox regression analysis was performed to evaluate factors potentially affecting grade ≥ 3 AEs. Subsequently, all covariates were further analyzed using a multivariate logistic regression model to confirm predictive factors. Statistical significance was considered at a *P*-value of < .05.

## Results

### Patient characteristics

**Figure 2.**
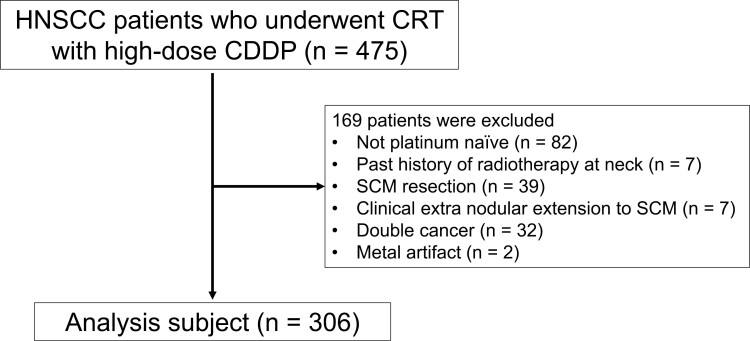
Patient selection criteria. A flowchart illustrating the composition of the study cohort (*n* = 306). Abbreviations: CDDP, cisplatin, CRT, chemoradiotherapy, HNSCC, head and neck squamous cell carcinoma, SCM, sternocleidomastoid muscle.

Overall, 475 consecutive patients underwent CRT with high-dose CDDP for locally advanced HNSCC at Shizuoka Cancer Center between January 2010 and April 2023. Among them, 82 patients with prior platinum chemotherapy, 7 with prior radiotherapy for neck lesions, 39 with prior SCM resection, 7 with clinical extra-nodal extension to the SCM, 32 with double malignancies, and 2 with metal artifacts on CT scans were excluded from the study. A final cohort of 306 patients were included in this study (Figure 2).

Patient characteristics are summarized in Table 1. The median age of the patients was 63 years (range, 23-78 years). Most patients (179/306, 58.5%) had a body mass index (BMI) of < 22.5 kg/m^2^. All patients had an ECOG PS score of 0-1. The primary tumor sites included the oral cavity in 78 (25.5%), hypopharynx in 73 (23.9%), larynx in 65 (21.2%), and oropharynx in 54 (17.6%) patients. A total of 242 patients (79.1%) were diagnosed with stages III and IV cancers. Most patients (70.8%) with stage I were oropharyngeal cancers tested positive for p16 immunohistochemistry,^21,22^ as per the Union for International Cancer Control 8th edition criteria. Definitive CRT was performed in 213 patients (69.6%), whereas 93 patients (30.4%) underwent postoperative CRT.

**Table 1.** Overall patient characteristics (n = 306)

Factors	Category	Patients (*n* = 306)
Sex, *n* (%)	Male	258 (84.3)
	Female	48 (15.7)
Age, *n* (%)	<60 years	107 (35.0)
	60≤ < 70 years	142 (46.4)
	≥70 years	57 (18.6)
Height (m)	Mean	1.66
	Range	1.37-1.82
Body weight (kg)	Mean	60.4
	Range	36.4-90.4
BMI (kg/m^2^), *n* (%)	≥22.5 kg/m^2^	127 (41.5)
	<22.5 kg/m^2^	179 (58.5)
PS (ECOG), *n* (%)	0	234 (76.5)
	1	72 (23.5)
UICC 8th stage, *n* (%)	I	24 (7.8)
	II	40 (13.1)
	III	56 (18.3)
	IV	186 (60.8)
Tumor site, *n* (%)	Oral cavity	78 (25.5)
	Hypopharynx	73 (23.9)
	Larynx	65 (21.2)
	Oropharynx	54 (17.6)
	Other^*^	36 (11.8)
Treatment setting, *n* (%)	Definitive	213 (69.6)
	Postoperative	93 (30.4)

^*^Others: nasopharynx, thyroid, salivary glands, nose, maxillary sinus, external auditory canal, and unknown primary.

Abbreviations: BMI, body mass index, ECOG, Eastern Cooperative Oncology Group, PS, performance status,. UICC, Union for International Cancer Control.

### Distribution of CDDP-related AEs according to the CDDP index values

**Figure 3.**
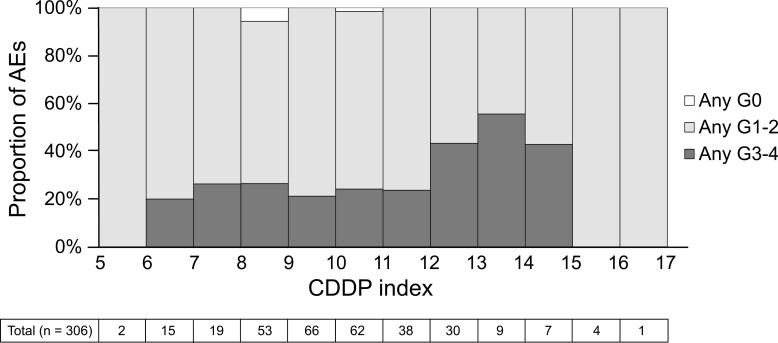
Distribution of grade ≥ 3 CDDP-related adverse events according to the CDDP index. Abbreviations: AEs, adverse events (hematological toxicities, gastrointestinal toxicities, nephrotoxicity, neuropathy, and infection), CDDP, cisplatin.

We investigated the prevalence and grading of CDDP-related AEs in patients with varying CDDP index values (Figure 3). For instance, among patients with CDDP index value 13.0-14.0, 5 out of 9 patients (55.6%) experienced grade ≥ 3 AEs, whereas among patients with CDDP index value 6.0-7.0, 3 out of 15 patients (20%) experienced grade ≥ 3 AEs. None of the patients with CDDP index value ≥ 15 (*n* = 5) and < 6 (*n* = 2) experienced grade ≥ 3 AEs, although the sample size in each category was small. The cutoff value for CDDP index best associated with grade ≥ 3 AEs was 10.312 based on ROC curve analysis (sensitivity = 0.553, specificity = 0.603, data not shown).

### Patient characteristics in the low CDDP index vs high CDDP index group

Using this cutoff value of CDDP index, we compared the clinical characteristics of patients with high CDDP index value and those with low CDDP index value who underwent CRT (Table 2). Patients aged < 60 years were more prevalent in the high CDDP index group than in the low CDDP index group. The mean BMI of patients in the high CDDP index group was significantly lower than that of patients in the low CDDP index group. Furthermore, baseline CCR calculated based on the Cockcroft-Gault equation was significantly higher in the high CDDP index group than in the low CDDP index group. CDDP dose of 100 mg/m^2^ was more frequently administered to patients in the high CDDP index group than to those in the low CDDP index group.

**Table 2.** Patient characteristics in the low CDDP index and high CDDP index groups

Factors	Category	Low CDDP index (n = 172)	High CDDP index (n = 134)	*p*-value^*^
Sex, *n* (%)	Male	147 (85.5)	111 (82.8)	0.531
	Female	25 (14.5)	23 (17.2)	
Age, *n* (%)	<60 years	43 (25.0)	64 (47.8)	<0.001
	60≤ < 70 years	88 (51.2)	54 (40.3)	
	≥70 years	41 (23.8)	16 (11.9)	
BMI (kg/m^2^)	Mean	22.5	21.5	0.014
	Range	13.9-34.3	14.5-31.1	
ECOG PS, *n* (%)	0	132 (76.7)	102 (76.1)	0.893
	1	40 (23.3)	32 (23.9)	
UICC 8th stage, *n* (%)	I	10 (5.8)	14 (10.4)	0.337
	II	25 (14.5)	15 (11.2)	
	III	29 (16.9)	27 (20.1)	
	IV	108 (62.8)	78 (58.3)	
Treatment setting	Postoperative	53 (30.8)	40 (29.2)	0.901
	Definitive	119 (69.2)	94 (70.1)	
Tumor site, *n* (%)	Oral cavity	42 (24.5)	36 (26.9)	0.736
	Hypopharynx	41 (23.8)	32 (23.9)	
	Larynx	41 (23.8)	24 (17.9)	
	Oropharynx	30 (17.4)	24 (17.9)	
	Other	18 (10.5)	18 (13.4)	
Albumin (g/dL)	Mean	4.06	4.08	0.709
	Range	3.1-5.2	3.0-5.0	
CCR (mL/minute)	Mean	86	98	<0.001
	Range	49-185	53-233	
CRP (mg/dL)	Mean	0.59	0.57	0.895
	Range	0.02-7.54	0.01-10.77	
CDDP/BSA, *n* (%)	80 mg/m^2^	125 (72.7)	30 (22.4)	<0.001
	100 mg/m^2^	47 (27.3)	104 (77.6)	
2nd cycle of CDDP	Continue	125 (72.7)	84 (62.7)	0.369
	Prolongation	16 (9.3)	15 (11.2)	
	Reduction	20 (11.6)	25 (18.6)	
	Interruption	4 (2.3)	3 (2.2)	
	Switch^**^	7 (4.1)	7 (5.3)	

^*^Fisher’s exact test or Student’s *t* test.

^**^Switch: switched to any other chemotherapy.

Abbreviations: BMI: body mass index, BSA: body surface area, CCR: creatinine clearance, CDDP: cisplatin, CRP: c-reactive protein,; ECOG: Eastern Cooperative Oncology Group, PS: performance status, UICC: Union for International Cancer Control.

### CDDP-related AE profile in the low CDDP index vs high CDDP index group

We compared the frequency of AEs between the low and high CDDP index groups (Table 3). The most prevalent AE was hematological toxicities in both the groups. Grade ≥ 3 hematological toxicities occurred significantly more frequently in the high CDDP index group than in the low CDDP index group (26.9% vs 16.3%, *P* = .033). However, no significant differences in the frequency of grade ≥ 3 gastrointestinal toxicities, nephrotoxicity, neuropathy, and infection were observed between the groups. Regarding all-grade toxicities, gastrointestinal toxicities occurred more frequently in the high CDDP index group than in the low CDDP index group (45.5% vs 31.9%, *P* = .018). The high CDDP index group exhibited a trend toward a high incidence of nephrotoxicity and neuropathy (*P* = .094 and *P* = .061, respectively). No association was observed between CDDP index and treatment compliance of second cycle of CDDP (Table 2).

**Table 3.** Adverse event profile of patients in the low CDDP index and high CDDP index groups.

Category	Grades 1-4, *n* (%)			Grade ≥ 3, *n* (%)		
	Low CDDP index (*n* = 172)	High CDDP index (*n* = 134)	*P-*value^*^	Low CDDP index (*n* = 172)	High CDDP index (*n* = 134)	*P*-value^*^
Hematological toxicities	156 (90.7)	125 (93.3)	0.529	28 (16.3)	36 (26.9)	0.033
Gastrointestinal toxicities	55 (31.9)	61 (45.5)	0.018	6 (3.5)	7 (5.2)	0.571
Nephrotoxicity	24 (14.9)	29 (21.6)	0.094	0 (0)	2 (1.5)	0.191
Neuropathy	60 (34.9)	61 (45.5)	0.061	0 (0)	0 (0)	1
Infection	6 (3.5)	3 (2.2)	0.736	6 (3.5)	3 (2.2)	0.736

^*^Fisher’s exact test.

Abbreviation: CDDP, cisplatin.

### Predictive factors associated with grade ≥ 3 CDDP-related AEs in univariate and multivariate analyses

Regarding predictive factors associated with grade ≥ 3 CDDP-related AEs, univariate analysis revealed that high CDDP index value and low CCR (< 70mL/minute) were significantly associated with the occurrence of grade ≥ 3 AEs (odds ratio [OR] 1.78, *P* = .027, OR 3.04, *P* < .001, respectively; Table 4). To adjust for significant predictive factors, a multivariate logistic regression model that included all factors was used. High CDDP index value (OR 2.55, *P* = .008) and low CCR (< 70 mL/minute) (OR 3.68, *P* = .002) were confirmed as independent predictive factors for grade ≥ 3 AEs. However, the predictive value of CDDP dose per BSA was not statistically significant (OR 1.47, *P* = .286). Elderly patients (60 years ≤ < 70 years, ≥ 70 years), low CRP level and high Charlson risk index were marginally associated with grade ≥ 3 AEs (OR 2.03, *P* = .057, OR 2.32, *P* = .086, OR 2.39, *P* = .069; OR 1.99, *P* = .057, respectively).

**Table 4.** Predictive factors associated with any grade ≥ 3 AEs in univariate and multivariate analysis.

Factors	Category	Any grade 0-2 (*n* = 225)	Any grade ≥ 3 (*n* = 81)	Univariate analysis		Multivariate analysis	
				OR (95% CI)	*P*-value	OR (95% CI)	*P*-value
Age, n (%)	<60 years	86 (38.3)	21 (25.9)	Ref.		Ref.	
	60≤ < 70 years	100 (44.4)	42 (51.9)	1.72 (0.946-3.130)	0.075	2.030 (0.978-4.230)	0.057
	≥70 years	39 (17.3)	18 (22.2)	1.89 (0.907-3.940)	0.089	2.320 (0.889-6.050)	0.086
Sex, *n* (%)	Female	35 (15.6)	13 (16)	Ref.		Ref.	
	Male	190 (84.4)	68 (84)	0.964 (0.464-2.108)	1.000	0.968 (0.446-2.100)	0.934
ECOG PS, *n* (%)	0	178 (79.1)	56 (69.1)	Ref.		Ref.	
	1	47 (20.9)	25 (30.9)	1.688 (0.910-3.091)	0.092	1.500 (0.764-2.930)	0.240
Treatment setting, *n* (%)	Postoperative	67 (29.8)	26 (32.1)	Ref.		Ref.	
	Definitive	158 (70.2)	55 (67.9)	0.897 (0.519-1.550)	0.697	0.847 (0.442-1.630)	0.618
Albumin, *n* (%)	≥3.5 g/dL	211 (93.8)	73 (90.1)	Ref.		Ref.	
	<3.5 g/dL	14 (6.2)	8 (9.9)	1.649 (0.575-4.416)	0.316	1.470 (0.496-4.350)	0.488
CRP, *n* (%)	<1 mg/dL	185 (83)	70 (88.6)	Ref.		Ref.	
	≥1 mg/dL	38 (17)	9 (11.4)	0.627 (0.253-1.406)	0.281	0.418 (0.163-1.070)	0.069
NLR, *n* (%)	<3.7	191 (86.8)	67 (87)	Ref.		Ref.	
	≥3.7	29 (13.2)	10 (13)	0.983 (0.455-2.120)	0.965	1.120 (0.461-2.730)	0.801
CCR, *n* (%)	≥80 mL/minute	147 (65.3)	43 (53.1)	Ref.		Ref.	
	70≤ < 80 mL/minute	51 (22.7)	14 (17.3)	0.938 (0.474-1.860)	0.855	0.892 (0.405-1.960)	0.776
	<70 mL/minute	27 (12.0)	24 (29.6)	3.040 (1.590-5.800)	<0.001	3.680 (1.620-8.360)	0.002
CDDP index, *n* (%)	<10.312	135 (60)	37 (45.7)	Ref.		Ref.	
	≥10.312	90 (40)	44 (54.3)	1.78 (1.070-2.980)	0.029	2.550 (1.270-5.100)	0.008
CDDP/BSA, *n* (%)	80 mg/m2	118 (52.4)	37 (45.7)	Ref.		Ref.	
	100 mg/m2	107 (47.6)	44 (54.3)	1.31 (0.764-2.258)	0.303	1.470 (0.725-2.980)	0.286
Charlson risk index, *n* (%)	2	183 (81.3)	60 (74.1)	Ref.		Ref.	
	≥3	42 (18.7)	21 (25.9)	1.523 (0.791-2.874)	0.199	1.990 (0.981-4.030)	0.057

Abbreviations: BSA, body surface area, CCR, creatinine clearance, CDDP, cisplatin, CI, confidence interval, CRP, c-reactive protein; ECOG, Eastern Cooperative Oncology Group, NLR, neutrophil-to-lymphocyte ratio, OR, odds ratio, PS, performance status, Ref., reference.

## Discussion

Our study investigated predictive factors associated with grade ≥ 3 CDDP-related adverse AEs in patients with HNSCC. We observed that high CDDP index values and low CCR were independently associated with an increased risk of grade ≥ 3 AEs. Additionally, elderly patients, low CRP levels and high Charlson risk index showed marginal associations with these adverse events. However, the predictive value of CDDP dose per BSA was not significant.

Both severe hematological and non-hematological AEs induced by CDDP during CRT can lead to unplanned treatment interruptions and prolonged overall treatment time, ultimately affecting therapeutic outcomes, including cure rates, remission durability, and survival.^23-25^ Furthermore, ototoxicity related to CDDP is irreversible and considerably worsen the quality of life in patients undergoing CRT.^26,27^ A previous study has demonstrated great variability in body composition among patients with cancer, and sarcopenia is reported as an independent risk factor for chemotherapy-associated toxicities in patients with respiratory and gastrointestinal malignancies.^18^ Therefore, we focused on body composition in patients with HNSCC and hypothesized that optimal dose settings based on SMM is crucial, particularly for patients with low SMM.

Several studies have reported an association between low SMM and an increased risk of chemotherapy-associated toxicities and subsequent poor treatment tolerability in head and neck cancer.^10,14,28^ We further analyzed the impact of the relative CDDP dose to C3-SMM on grade ≥ 3 CDDP-related AEs in patients with HNSCC undergoing CRT with high-dose CDDP. The CDDP index was originally defined as the prescribed CDDP dose divided by C3-SMI, which is the C3-SMM normalized for squared height. The results revealed that a high CDDP index value is an independent predictive factor for grade ≥ 3 AEs. This is consistent with previous studies in which relative dose of fluoropyrimidine to estimated LBM had impact on chemotherapy-associated toxicities in breast and colorectal cancers.^4,19,29^ Our results highlight the critical role of C3-SMM assessment in determining CDDP dosage. Association between low SMM and increased toxicities induced by CRT was reported even in patients with locally advanced HNSCC treated with low-dose weekly CDDP-based chemotherapy.^30^ This finding also supports a tailored approach to chemotherapy dosing based on the individual patient’s SMM.

BSA-based dosing in medical oncology lacks strong evidence correlating interpatient pharmacokinetic with BSA.^31,32^ However, previous studies have demonstrated a weak association between estimated LBM and BSA in breast, gastrointestinal, and respiratory cancers.^4,18,32^ Consequently, considerable interpatient variations in dose occur when expressed per kilogram of LBM.^4,18^ Low LBM would be a low volume of distribution of cytotoxic chemotherapy drugs and previous study mentioned that higher doses of fluorouracil per kilogram LBM was associated with a higher incidence of overall toxicity.^19^ Our results also suggest that high CDDP index value was a more potent predictive factor for grade ≥ 3 AEs than CDDP dose per BSA. These findings suggest that the BSA-based dosing of chemotherapeutic agents may lead to overdosing in patients with relatively low SMM but high body fat mass.^33-35^ Indeed, a retrospective cohort study revealed that sarcopenia was associated with a low relative dose intensity of perioperative chemotherapy in patients with locally advanced esophageal and colorectal cancers,^8,36^ possibly due to initial BSA-based dosing resulting in overdoses, necessitating subsequent dose modifications in patients with sarcopenia. Dose adjustment of CDDP may mitigate severe AEs associated with high-dose CDDP in patients with high CDDP index value, whereas dose escalation of CDDP may be tolerable to maintain dose intensity in patients with low CDDP index value. Consequently, CDDP index may be a novel useful marker for predicting toxicities induced by BSA-based CDDP dosing and better individualizing CDDP doses according to SMM.

CISLOW study is currently ongoing, in which the patients with low SMM will be randomized into either weekly low dose CDDP or triweekly high dose CDDP.^37^ If the result is positive, patients with low SMM might benefit from weekly low dose CDDP-based CRT to achieve an adequate cumulative dose. Although the focus of our study is CDDP-related AEs in patients treated by high-dose CDDP, weekly low dose CDDP may be an alternative option in patients with low SMM.

To further characterize patients with high and low CDDP index values, we investigated the clinical characteristics of patients with HNSCC with respect to CDDP index values. The high CDDP index group was significantly associated with a lower BMI, indicating that an overdose of CDDP was administered to patients with low SMM. Moreover, we observed a statistically significant correlation between high CCR and high CDDP index values. This finding may be partially explained by the fact that a higher dose of CDDP per SMI is administered to patients with favorable CCR compared to those with poor renal function in clinical practice.

Our study cohort includes both patients who received definitive CRT and postoperative CRT. However, a criticism may occur that surgery for head and neck cancer can lead to significant loss of SMM due to reduced physical activity and systemic inflammatory responses. Indeed, SMM in patients who received postoperative CRT was significantly smaller than that in those who did definitive CRT (*P* = .0158) (Supplementary figure). However, multivariate analysis revealed that postoperative setting had no impact on CDDP-related toxicities (OR 0.847, *P* = .618; Table 4), suggesting that presence or absence of prior surgical invasion was not an independent predictive factor for AEs. The CT scan for radiotherapy planning after surgery was mostly used as the baseline CT in patients who received postoperative CRT. Therefore, surgery-induced SMM loss was taken into consideration in patients who received postoperative CRT in our cohort.

CT scans have proven to be accurate for measuring human body composition and SMM.^38-40^ Although SMM measurement using a single CT slide at the third lumbar vertebra (L3) is the standard radiological assessment method for sarcopenia,^4-8^ imaging at L3 is not routinely performed in clinical practice for patients with HNSCC. Swartz et al. reported a strong correlation between C3-SMM and L3-SMM in patients with head and neck cancer.^41^ Therefore, C3-SMM measurement may be reliable for estimating body composition and was alternatively used in our study.

This study has a few limitations. First, it was a retrospective study conducted at a single institution. However, we included over 300 consecutive patients between 2010 and 2023. To confirm the utility of the CDDP index, further validation is required using an independent dataset of patients who underwent CRT. Second, there were variations in the period between the timing of CT scan and treatment initiation, which may at least partially influence the nutritional status and SMM of patients. Third, calibration between two radiologists was not performed to confirm SMM in our study. Finally, this study did not focus on the correlation between CDDP index and long-term treatment outcomes.

## Conclusion

Our analysis suggested that CDDP index based on C3-SMM would be an independent predictive factor for grade ≥ 3 CDDP-related AEs. C3-SMM may be a more useful index than BSA for determining the optimal CDDP dose in patients with HNSCC. Therefore, further evaluation of C3-SMM is needed in the dose setting of high-dose CDDP in CRT for patients with HNSCC.

## Supplementary material

Supplementary material is available at *The Oncologist* online.

oyae167_suppl_Supplementary_Figure

## Data Availability

The data that support the findings of this study are available from the author, upon reasonable request.
