# Supplementary material for: Impact of relative cisplatin dose to skeletal muscle mass on adverse events in patients with head and neck cancer undergoing chemoradiotherapy
Source: Oncologist. 2024 Jul 9;29(10):e1315–23. doi: 10.1093/oncolo/oyae167 (PMC11449007; doi:10.1093/oncolo/oyae167)
Supplement: oyae167_suppl_Supplementary_Figure [file oyae167_suppl_supplementary_figure.pptx]

## Slide 1
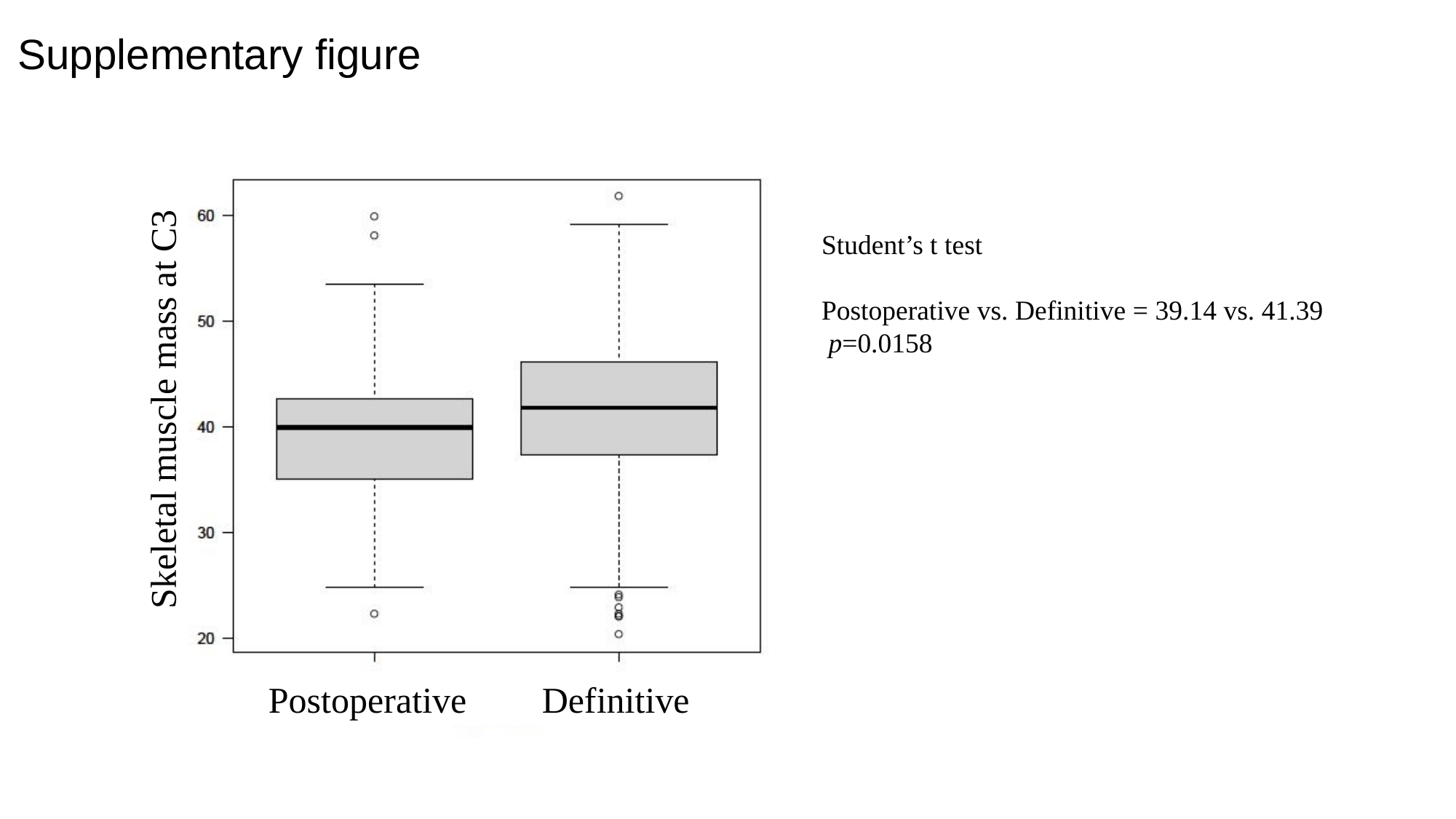

Supplementary figure
Skeletal muscle mass at C3
Definitive
Postoperative
Student’s t test
Postoperative vs. Definitive = 39.14 vs. 41.39
 p=0.0158
